# Upregulation of lnc-FOXD2-AS1, CDC45, and CDK1 in patients with primary non-M3 AML is associated with a worse prognosis

**DOI:** 10.1007/s44313-024-00002-0

**Published:** 2024-02-19

**Authors:** Saba Manoochehrabadi, Morteza Talebi, Hossein Pashaiefar, Soudeh Ghafouri-Fard, Mohammad Vaezi, Mir Davood Omrani, Mohammad Ahmadvand

**Affiliations:** 1 Department of Medical Genetics, Faculty of Medicine, Shahid Beheshti University of Medical Sciences, Tehran, Iran; 2 Department of Medical Genetics and Molecular Biology, Faculty of Medicine, Iran University of Medical Sciences, Tehran, Iran; 3 Cell Therapy and Hematopoietic Stem Cell Transplantation Research Center, Hematology and Cell Therapy, Research Institute for Oncology, Tehran University of Medical Sciences, Tehran, Iran

**Keywords:** Acute myeloid leukemia, Bioinformatics, Lnc -FOXD2-AS1, CDC45, CDK1, CDC20 and CCNB1

## Abstract

Acute myeloid leukemia (AML) is a heterogeneous hematologic malignancy with an unfavorable outcome. The present research aimed to identify novel biological targets for AML diagnosis and treatment. In this study, we performed an in-silico method to identify antisense RNAs (AS-RNAs) and their related co-expression genes. GSE68172 was selected from the AML database of the Gene Expression Omnibus and compared using the GEO2R tool to find DEGs. Antisense RNAs were selected from all the genes that had significant expression and a survival plot was drawn for them in the GEPIA database, FOXD2-AS1 was chosen for further investigation based on predetermined criteria (logFC ≥|1| and *P* < 0.05) and its noteworthy association between elevated expression level and a marked reduction in the overall survival (OS) in patients diagnosed with AML. The GEPIA database was utilized to investigate FOXD2-AS1-related co-expression and similar genes. The Kyoto Encyclopedia of Genes and Genomes (KEGG) pathway enrichment analysis and gene ontology (GO) function analysis of the mentioned gene lists were performed using the DAVID database. The protein–protein interaction (PPI) network was then constructed using the STRING database. Hub genes were screened using Cytoscape software. Pearson correlation analysis was conducted using the GEPIA database to explore the relationship between FOXD2-AS1 and the hub genes. The transcription of the selected coding and non-coding genes, including FOXD2-AS1, CDC45, CDC20, CDK1, and CCNB1, was validated in 150 samples, including 100 primary AML non-M3 blood samples and 50 granulocyte colony stimulating factor (G-CSF)-mobilized healthy donors, using quantitative Real-Time PCR (qRT-PCR). qRT-PCR results displayed significant upregulation of lnc-FOXD2-AS1, CDC45, and CDK1 in primary AML non-M3 blood samples compared to healthy blood samples (*P* = 0.0032, *P* = 0.0078, and *P* = 0.0117, respectively). The expression levels of CDC20 and CCNB1 were not statistically different between the two sets of samples (*P* = 0.8315 and *P* = 0.2788, respectively). We identified that AML patients with upregulation of FOXD2-AS1, CDK1, and CDC45 had shorter overall survival (OS) and Relapse-free survival (RFS) compared those with low expression of FOXD2-AS1, CDK1, and CDC45. Furthermore, the receiver operating characteristic (ROC) curve showed the potential biomarkers of lnc -FOXD2-AS1, CDC45, and CDK1 in primary AML non-M3 blood samples. This research proposed that the dysregulation of lnc-FOXD2-AS1, CDC45, and CDK1 can contribute to both disease state and diagnosis as well as treatment. The present study proposes the future evolution of the functional role of lnc-FOXD2-AS1, CDC45, and CDK1 in AML development.

**Supplementary Information:**

The online version contains supplementary material available at 10.1007/s44313-024-00002-0.

## Introduction

Acute myeloid leukemia (AML) is a set of blood-related cancers that involve the aberrant growth of myeloid blasts in the bone marrow (BM) and bloodstream. This growth leads to difficulties with the production of healthy blood cells [1]. AML pathophysiology can be mainly described by cytogenetic aberrations, gene mutations, and aberrant gene expression [2]. The standard treatments for the disease are determined by the particular subgroup of the illness that a patient has, and these can involve the use of induction chemotherapy as well as transplantation of hematopoietic stem cells. [3]. Presently established risk categorization recommendations demonstrate a modest predictive accuracy, and recent stratification that combine several molecular aberrations have proven to provide improved results.Currently determined AML risk categorization recommendations, such as the European Leukemia Net (ELN) risk stratification, are based mainly on a restricted number of molecular and cytogenetic aberrations. However, these recommendations don’t take into account the entire mutational profile of AML, the different levels of biological complexity in the cancer, the complex intertwining between AML patients, and the complexity of molecular interactions. Hence, there is considerable scope to enhance the prediction of AML survival [4]. Cytogenetic aberrations correlated with AML are documented as being the most prominent prognostic markers. However, approximately 40–50% of patients with AML are cytogenetically normal (CN-AML)**,** which makes it challenging to determine the prognosis for this group. In these cases, there are no acceptable biomarkers to determine the disease prognosis [5]. The risk stratification of CN-AML should be evaluated only based on molecular aberrations due to normal cytogenetic characteristics. Furthermore, patient clinical outcomes in this subgroup are also varied and challenging to describe [6]. However, heterogeneity in prolonged AML outcomes is an ongoing subject, and there is a need to identify novel biomarkers that can improve diagnosis and precise prognostication. ELN and WHO have accepted the role of gene expression in predicting the prognosis of AML patients; therefore, today, gene transcription and bioinformatics investigation are widely used to explain the underlying molecular mechanisms of several diseases [7]. Since several genes are involved in the pathway of tumorigenesis and these genes can interact with each other and act via a regulatory network; thus, it is very significant to evaluate the correlation between biomarkers and AML patients at the genetic and protein levels and clinical markers. Recently, the technology of gene chip has acted a significant role in exploring cancer gene transcription profiles and searching for cancer crucial genes [8]. Therefore, we tried to use bioinformatics tools to identify genes whose changes in the peripheral blood and in early disease stages can help in AML diagnosis and treatment. The regulation of normal hematopoiesis is dependent on strongly regulated and interrelated mechanisms that control cell proliferation and differentiation. Changes in the pathways of cell cycles are linked to the development of leukemia [9].The interaction of deregulated proliferation and differentiation processes in turn has significant effects on the changed control of cell cycle regulators, such as checkpoint kinases, cyclin-dependent kinases (CDKs), and mitotic kinases [10]. According to the importance of genes involved in the cell cycle, including cell division cycle 45 (CDC45)**,** cyclin-dependent kinase 1 (CDK1)**,** cell division cycle 20 (CDC20), and cyclin B1 (CCNB1), we planned the present work to identify long non-coding RNAs (lncRNAs) that have co expression with these genes via a systems biology approach. Then, we evaluated the transcription of lncRNA-FOXD2 adjacent opposite strand RNA 1 (FOXD2-AS1)**,** CDC45, CDC20, CDK1, and CCNB1 genes in primary AML non-M3 blood samples versus granulocyte colony stimulating factor (G-CSF)-mobilized healthy blood samples. This study aims to provide a novel way for early diagnosis and personalized treatment at the gene level.

## Materials and methods

### Selection of differentially expressed antisense RNA in primary AML using the GEO database

An in-silico investigation was conducted using the GEO (Gene Expression Omnibus) database to define differentially expressed genes (DEGs) in primary AML vs. healthy controls. The GSE68172 dataset, containing 72 primary AML blood samples and 5 healthy controls, was obtained. Of the numerous AML transcription profiling studies, this particular study was chosen because of the resemblance of its sample type to our own, namely blood samples and not bone marrow. In addition, the primary AML samples were more congruent with our research objectives. The mentioned dataset was downloaded using the "GEOquery" package in R software (3.5.1). DEGs screening process was performed using the "limma" package in R software (3.5.1) [13]. FOXD2-AS1 was identified for in-depth exploration based on the following stringent criteria: a fold change greater than 1 and an adjusted p-value less than 0.05. To elucidate our rationale, aligning with the study’s objectives, we initially extracted significantly over-expressed genes from GSE68172. Subsequently, antisense RNAs were scrutinized from this gene pool, with FOXD2-AS1 and IL10RB-AS1 emerging as the most prominently differentially expressed antisense RNAs in AML. Given FOXD2-AS1's notable association with critical cellular pathways, such as cell cycle, we opted to delve deeper into its potential as a biomarker for AML. Overall survival analysis was performed using GEPIA2 webserver to predict the prognostic values of FOXD2-AS1 in patients with primary non-M3 AML. In addition, literature review has shown that this novel lncRNA has an impact on the tumorigenesis of other human cancers.

### Investigating co-expressed genes associated with FOXD2-AS1

Gene Expression Profiling Interactive Analysis (GEPIA) was executed in order to investigate the genes that exhibit similarity or co-expression with FOXD2-AS1. The Protein–protein interactions** (**PPI) network of the above-mentioned genes was constructed using STRING, and was subjected to a threshold of moderate confidence (> 0.4) for interactions. Interactions were visualized using Cytoscape software. CytoHubba plugin was used to screen the top 10 hub genes based on the Matthews correlation coefficient (MCC) algorithm.

### Gene expression correlation analysis

To assess a conceivable correlation between FOXD2-AS1 and the hub genes, Pearson pairwise examination of gene expression correlation was performed based on the TCGA-LAML patient cohort available in the GEPIA. Significant relevance was defined by a correlation coefficient (R) (> 2 and < -2) and *P*-Val < 0.05.

### Functional analyses

GO annotation, including molecular function, biological pathway, cell component, and KEGG, was used to clarify the potential function of FOXD2-AS1-related hub genes in tumorigenesis. Functional analysis was conducted using the Enrichr online tool (https://maayanlab.cloud/Enrichr/).

### Survival analysis

To explore the impact of FOXD2-AS1 on overall survival (OS), the online tool GEPIA2 was used (http://gepia2.cancer-pku.cn/). To conduct the analysis, we used the TCGA-LAML dataset and applied the default parameters.

### Patients

One hundred non-M3 AML cases, diagnosed based on the French–American–British* (*FAB) and World Health Organization (WHO) criteria at the Research Institute for Oncology, Hematology and Cell Therapy, Tehran, Iran, were included in this study. The control group included 50 volunteers who received G-CSF, consisting of 24 men and 26 women, with a median age of 37 years (range: 22–71 years). Non-M3 AML patients received standard induction chemotherapy consisting of daunorubicin (60 mg/m^2^ daily for 3 days) plus cytarabine (100 mg/m^2^ daily for 7 days), and consolidation chemotherapy with two-to-four courses of high-dose cytarabine (2000 mg/ m^2^ every twelve hours for 4 days, total eight doses), with or without an anthracycline (idarubicin or mitoxantrone), after complete remission was achieved. After the first remission, 12 patients underwent allogeneic hematopoietic stem-cell transplantation (HSCT).

This work was approved by the Shahid Beheshti University of Medical Science ethical committee (ethical code: IR.SBMU.MSP.REC.1401.302). informed consent to participate in this study was obtained from all participants.

### Quantitative Real-Time PCR

Quantitative Real-Time PCR (qRT-PCR) was performed in a LightCycler® 96 System (Roche) using SYBR Green methodology (ampliqon). To ensure the specificity of the primers, melting peak analysis was performed. To determine the efficiency of qRT-PCRs, a standard curve was constructed using a serial dilution (five point) of cDNA samples. The transcription levels of the target genes were normalized to those of the reference gene beta-2-microglobulin (B2M). Relative quantification was performed using the 2-^DCt^ method.

### Statistical analysis

Statistical analyses were applied using SPSS 20.0 software package. To compare the association of multiple variables, Pearson v2 analysis or Fisher’s exact test were employed. Kolmogorov–Smirnov tests were employed to check the variables’ normal distribution**.** Spearman rank correlation coefficient analysis was performed to explore the association between target gene transcription levels and clinicopathological features. Receiver operating characteristic curve (ROC) curve analyses were performed to evaluate the sensitivity and specificity for each value of the measure, and the area under the curve (AUC) was computed at a confidence interval of 95%, indicating the capability of the biomarker to distinguish between the two groups. OS was analyzed from the first diagnosis date to the death date of any cause, and RFS was analyzed from the complete remission (CR) date after initial therapy to the relapse date or death of any cause. Patients who received allogeneic HSCT were censored on the day of stem cell infusion. Survival curves for RFS and OS were estimated using Kaplan–Meier and their 95% confidence intervals (CI) were calculated using the log-rank method. The Cox proportional hazards model was used, adjusting for potential confounding covariates. For multivariate analysis, variables with a *p*-value of 0.2 or less were included in univariate analysis. Statistical significance was determined at a *p*-value of less than 0.05.

## Results

### The notable upregulation of FOXD2-AS1 expression level in primary AML, its diagnostic value, and its influence on patient survival are predicated on bioinformatics scrutiny

To identify antisense RNAs that possess oncogenic properties, the up-regulated genes were distinguished from the differentially expressed genes (DEGs). The over-expressed gene list was used to screen for antisense RNAs. Subsequently, the antisense RNAs with the most elevated logFC were meticulously filtered, and FOXD2-AS1 was ultimately chosen through evaluation of its functions. Based on the GSE68172 dataset, FOXD2-AS1 exhibited significant overexpression in primary AML samples compared to healthy control (as depicted in Supplementary Fig. 1A), with a logFC of 1.65190288 and a *p*-value of 2.52E-03. The analysis of the ROC curve further indicated a significant level of efficacy of FOXD2-AS1 as a diagnostic indicator, with an AUC value of 0.9389 and a *p*-value of 0.0011 (Supplementary Fig. 1B). The findings have illustrated that FOXD2-AS1 possesses the potential to function as a biomarker with the ability to effectively differentiate between primary AML cases and normal cases displaying oncogenic characteristics. Moreover, a survival analysis conducted on the TCGA-LAML dataset within the GEPIA2 platform revealed a noteworthy association between elevated FOXD2-AS1 expression levels and a marked reduction in the OS of patients diagnosed with AML. (Supplementary Fig. 1C).

### The genes CDC45, CDC20, CDK1, and CCNB1 exhibit positive co-expression with FOXD2-AS1 as established through bioinformatics analysis

To determine the genes that displayed affirmative co-expression with FOXD2-AS1, a total of 500 genes were retrieved from the GEPIA repository. The PPI network illustrated in Supplementary Fig. 2A and B encompasses the aforementioned genes. The MCC algorithm, within the Cytohabba plugin, identified CDC45, CDC20, KIF2C, KIF20A, TOP2A, CDK1, ASPM, AURKA, NCAPG, and CCNB1 as central hub genes (Supplementary Fig. 2C). Pairwise analysis of gene expression correlation revealed that CDC45(R = 0.48), CDK1(R = 0.38), CCNB1(0.35), and CDC20(0.33) exhibit the strongest positive co-expression with FOXD2-AS1, respectively (Supplementary Fig. 3A-D).

### Genes co-expressed with FOXD2-AS1 are implicated in critical cellular processes, including the regulation of the cell cycle

**Fig. 1:**
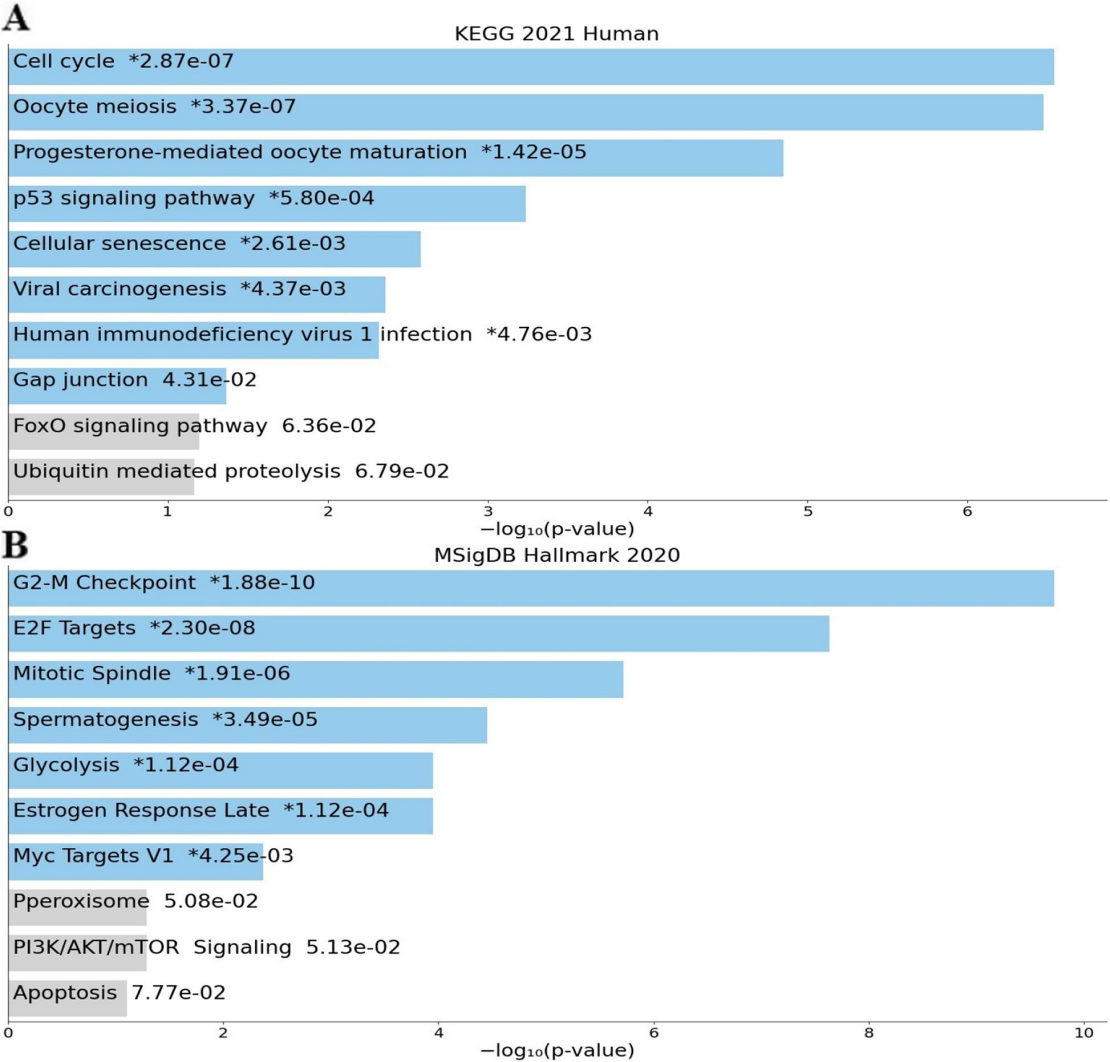
KEGG **A** and biological pathway analysis **B**

In order to ascertain the potential role of FOXD2-AS1-related genes, a functional analysis was conducted on hub genes that exhibited positive co-expression with FOXD2-AS1. Based on KEGG, the aforementioned hub genes are implicated in crucial pathways, including cell cycle regulation, the P53 signaling pathway, and viral carcinogenesis (Fig. 1A). Furthermore, a biological pathway analysis, based on the Molecular Signatures Database (MsigDB), revealed that the mentioned genes similarly influence cell cycle regulation through the G2M checkpoint, E2F targets, and mitotic spindle (Fig. 1B). GO annotation, encompassing molecular roles, cell components, and biological pathways, established that FOXD2-AS1, in collaboration with other proteins such as CDC20, CDC45, CDK1, KIF2C, KIF20A, TOP2A, ASPM, NCAPG, AURKA, and CCNB1, is enriched in molecular functions such as protein kinase binding. In addition, it may participate in the regulation of biological processes, cell cycle regulation, chromosome segregation, and the mitotic checkpoint **(**Supplementary Table 1a-c).

### Expression levels of FOXD2-AS1, CDC45, CDC20, CDK1, and CCNB1 in non-M3 AML patients

**Fig. 2:**
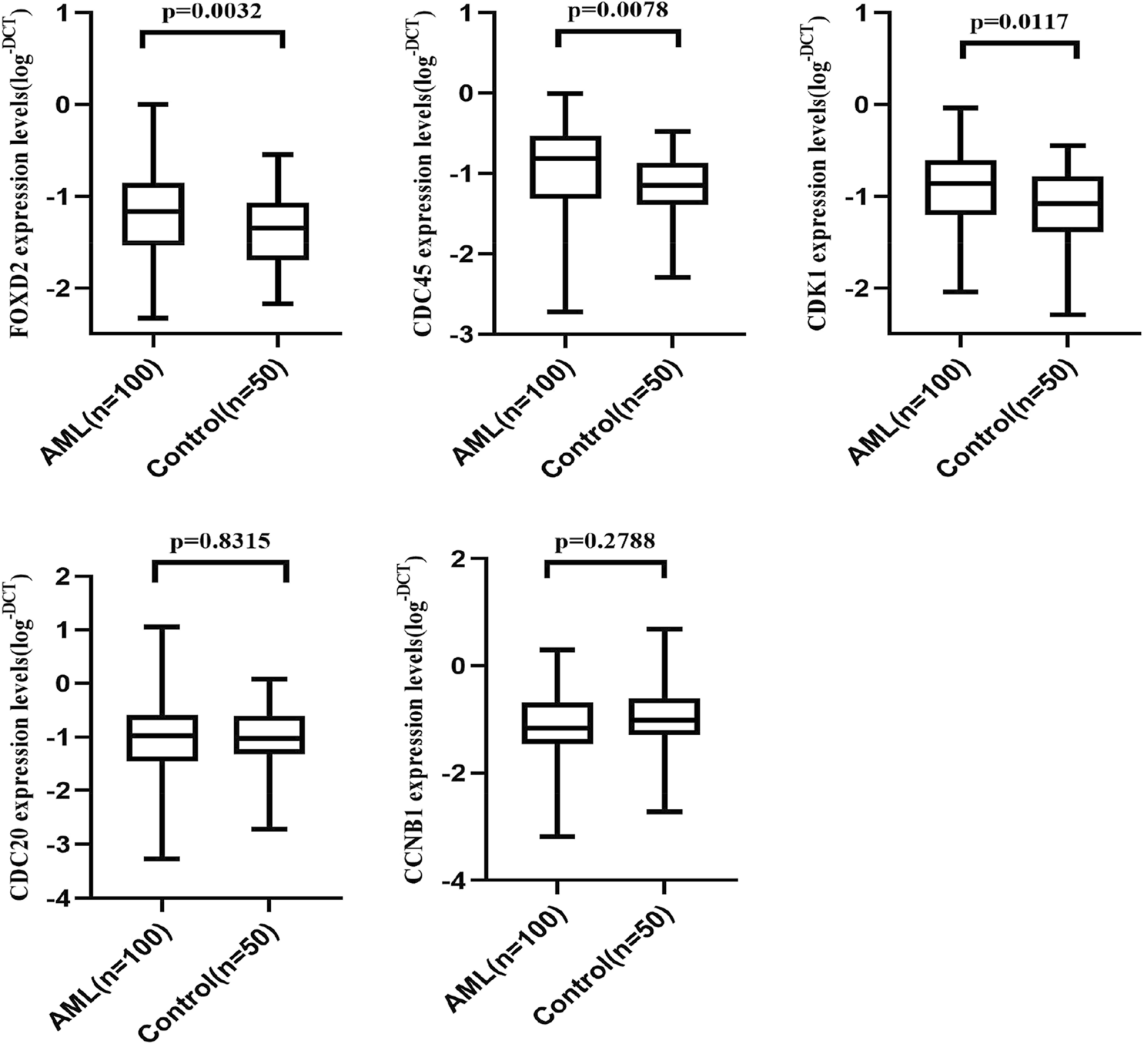
Relative expression in non-M3 AML cases and healthy groups detected by qRT-PCR analysis

The transcription levels of FOXD2-AS1, CDC45, CDC20**,** CDK1, and CCNB1 were quantified in blood from 100 Primary AML- non M3 patients and 50 G-CSF-mobilized healthy blood samples. The results showed that lnc- FOXD2-AS1, CDC45, and CDK1 were statistically upregulated in AML samples compared with the healthy group (*P* = 0.0032), (*P* = 0.0078), and (*P* = 0.0117), respectively (Fig. 2). Expression levels of CDC20, and CCNB1 were not statistically different between the two sets of samples (*P* = 0.8315 and *P* = 0.2788, respectively) (Fig. 2).

### Correlation between FOXD2-AS1, CDC45, and CDK1 transcription and clinicopathological features of non-M3 AML cases

The clinicopathological features of AML patients were assessed to investigate their associations with the high (≥ median value) or low (< median value) transcriptions of FOXD2-AS1, CDC45, and CDK1 in AML (Table 1). Upregulation of FOXD2-AS1 was significantly correlated with median WBC (*P* = 0.025) and BM blasts (*P* = 0.00054). Upregulation of CDC45 was significantly correlated with median WBC (*P* = 0.002), median hemoglobin (*P* = 0.005), and gene mutation (FLT3-ITD (+ /–) (*P* = 0.037). Additionally, upregulated CDK1 was significantly correlated with Median WBC (*P* = 0.005), median hemoglobin (*P* = 0.05) and karyotype classification (*P* = 0.02).

**Table 1:** Clinicopathological characteristics of Non-M3 AML cases and transcription status of FOXD2AS1, CDK1 and CDC45

Patients’ parameters	High FOXD2AS1 expression (*n* = 50)	Low FOXD2AS1 expression (*n* = 50)	*P* value	High CDK1 expression (*n* = 50)	Low CDK1 expression (*n* = 50)	*P* value	High CDC45 expression (*n* = 50)	Low CDC45 expression (*n* = 50)	*P* value
Sex, male/female	30/20	25/25	0.314879	25/25	30/20	0.314879	24/26	31/19	0.159412
Median age, years (range)	57 (16–75)	55 (20–68)	0.215444	55 (16–74)	56 (21–75)	0.602357	55 (16–75)	56 (20–74)	0.371669
Median WBC, × 10^9^/L (range)	64 (3–163)	48 (10–140)	0.025866*	59 (3–163)	48 (4–135)	0.005017*	65 (3–163)	44 (4–135)	0.002552*
Median hemoglobin, g/L (range)	8 (4–15)	8.3 (3.9–12)	0.436450	8 (3.9–15)	8.25 (4.6–14)	0.058183*	8 (4–12)	9 (5–15)	0.005790*
Median platelets, × 10^9^/L (range)	54.5 (4–302)	58.5 (4–190)	0.881138	55 (4–302)	56 (4–170)	0.746145	53 (4–302)	59 (4–190)	0.375813
BM blasts, % (range)	45.5 (12–90)	36 (20–92)	0.00054*	38 (12–90)	36 (18–92)	0.194970	38 (12–92)	36 (18–90)	0.587286
FAB, n (%)									
M0	9	9		8	10		10	8	
M1	13	15		18	10	0.214784	16	12	
M2	16	14	0.975619	13	17		13	17	0.735896
M4	8	9		6	11		7	10	
M5	4	3		5	2		4	3	
M6	-	-		-	-		-	-	
Karyotype classification, n (%)									
Favorable AND Intermediate	29	36	0.142213	27	38	0.021098*	30	35	0.294507
Unfavorable	21	14		23	12		20	15	
Gene mutation									
NPM1 (+ /–)	9/41	6/44	0.400814	6/44	9/41	0.400814	7/43	8/42	0.779435
FLT3-ITD (+ /–)	15/35	10/40	0.248213	8/42	17/33	0.37667	8/42	17/33	0.037667*
Response to treatment, n (%)									
CR	33	38	0.270506	36	35	0.825575	36	35	0.825575
NR	17	12		14	15		14	15	
Genetic risk (ELN)									
Favorable	13	14	0.1023	13	16	0.04120*	14	15	0.1785
Intermediate	19	20		17	20		18	19	
Poor	18	16		20	14		18	16	

^*^P < 0.05

### FOXD2-AS1 and related gene expression as potential diagnostic biomarkers of non-M3 AML

**Fig. 3:**
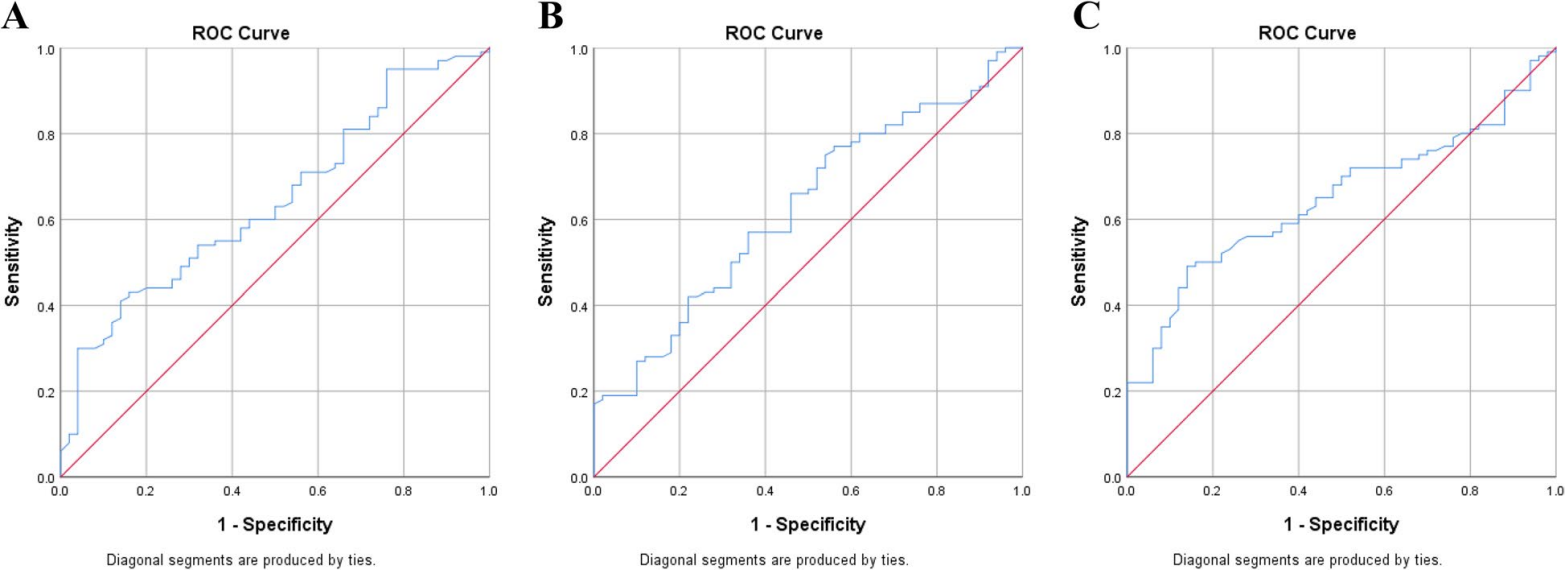
ROC curve analysis of lnc -FOXD2-AS1 and related genes as biomarkers to discriminate non-M3 AML patients from healthy controls. **A**, **B**, **C** The sensitivity and specificity of lnc -FOXD2-AS1, CDK1 and CDC45 transcription were 82% and 70% (AUC = 0.643, *P* = 0. 004). 72% and 50% (AUC = 0.62, *P* = 0.012). 45% and 78% (AUC = 0.64, *P* = 0.003) respectively

The potential diagnostic values of lnc FOXD2-AS1, CDK1, and CDC45 expression in AML were assessed by AUC from the ROC curve analysis (Fig. 3). The findings of this study recommended that lnc-FOXD2-AS1 expression could serve as a biomarker with a sensitivity of 82% and specificity of 70% (AUC = 0.643, *P* = 0. 004) (Fig. 3A). The sensitivity and specificity for the related genes CDC45 and CDK1 are also shown in Fig. 3B and C.

### High expression of FOXD2-AS1, CDK1, and CDC45 was a worse prognostic factor in patients with non-M3 AML

**Fig. 4:**
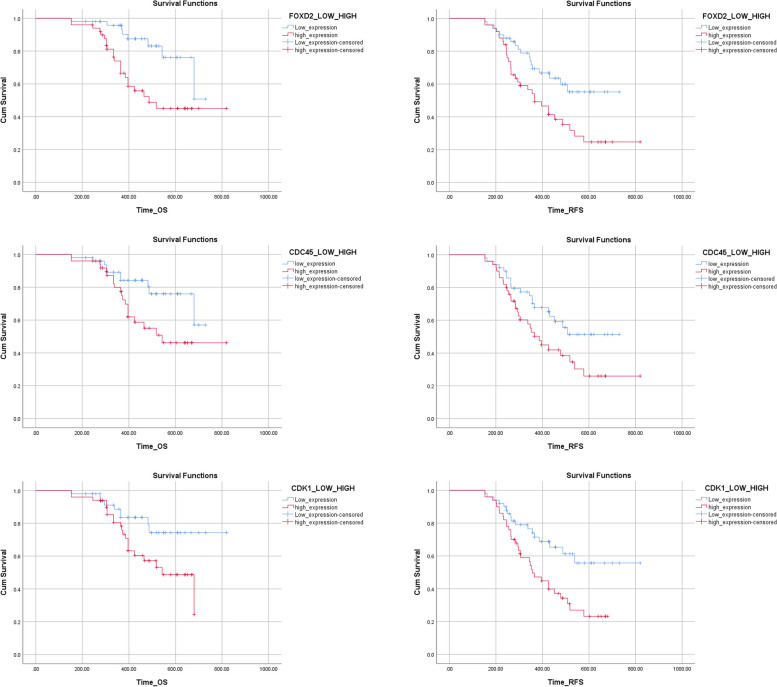
Kaplan–Meier curves of OS and RFS and of non-M3 AML cases stratified by the FOXD2-AS1, CDK1 and CDC45 expression levels. Patients with high FOXD2-AS1, CDK1 and CDC45 expression levels had shorter RFS and OS than those with low FOXD2-AS1 CDK1 and CDC45 transcription levels (*p* = 0.01 and 0.002, *p* = 0.05 and 0.02, *p* = 0.03 and 0.02, respectively)

To explore the prognostic effect of FOXD2-AS1, CDK1, and CDC45 high expression in non-M3 AML cases, OS analysis was performed in 100 patients with non-M3 AML. The median follow-up was 10.13 months ranging from 1 to 16.17 months. Kaplan–Meier curves for RFS and OS were classified based on FOXD2-AS1, CDK1, and CDC45 levels in patients with non-M3 AML. We identified that AML cases with upregulation of FOXD2-AS1, CDK1, and CDC45 had shorter OS and RFS than those with low levels of FOXD2-AS1, CDK1, and CDC45 expression (Fig. 4).

## Discussion

AML is a heterogeneous malignant clonal disorder of immature myeloid cells resulting in dysregulated normal hematopoiesis. [11]. It is well known that mutation in genes and chromosomes play a significant role in leukemogenesis [12]. The advent of microarray technology has quickly improved the volume of available genetic data [13]. In the present work, we established an in-silico approach to find antisense RNAs (AS-RNAs) and their related co-expression genes. This work used bioinformatic tools to identify DEGs in the AML dataset (GSE68172), which revealed upregulated and downregulated genes. Next, this study focused on upregulated DEG networks, as they are more likely to be diagnostic or prognostic biomarkers in AML. The dysregulated DEGs were mostly enriched in numerous pathways associated with nuclear division, p53 signaling pathway, cell cycle, cellular senescence, and DNA replication. Finally, it was found that Lnc-FOXD2-AS1 and CDK1, CCNB1, CDC20, and CDC45 genes were corelated to the worse survival of patients with AML. This work was confirmed at the expression level and evaluated the relationship between clinical characteristics and gene expression. It is becoming evident that numerous lncRNAs play a crucial role in controlling gene transcription during the development of blood cells; therefore, their deregulated transcription may lead to impairments in uncontrolled blood cell proliferation and differentiation, leading to leukemogenesis [14]. Research has demonstrated that certain transcripts may be an important tool for stratification of blood malignancy and have received growing attention in the leukemia field [15]. Expression analysis of the mentioned genes in primary AML non-M3 blood samples revealed overexpression of FOXD2-AS1 compared with G-CSF-mobilized healthy blood samples.

LncRNA FOXD2-AS1 is a promising biomarker among all cancer-related lncRNAs [16]. Recently, it was detected that FOXD2-AS1 is abnormally expressed in some different types of malignancy. This dysregulation may be correlated with the outcome of individuals with cancer. [16]. One study from Xu et al. showed that FOXD2-AS1 upregulation might be detected in gastric cancer tissues compared with adjacent non-cancerous tissues and was closely associated with tumor size and worse prognosis. Furthermore, another comprehensive study recognized that FOXD2-AS1 knockdown induced arrest in the G1 phase and reduction in the S phase, causing an important decrease in leukemogenicity, whereas FOXD2-AS1 upregulation increased cell proliferation. Altogether, FOXD2- AS1 can act as a key tumor-increasing gene in gastric cancer and can be a new biomarker and promising target for treatment [17]. Previous studies have shown that the upregulation of FOXD2-AS1 has a leading function in cancers and showed its carcinogenic potential by several mechanisms such as inducing cell division and metastasis. The manner in which competing endogenous RNA (ceRNA) is a crucial mechanism of FOXD2-AS1 in tumorgenicity. FOXD2-AS1 serves as a ceRNA for miR-7-5p and overexpresses the telomerase reverse transcriptase (TERT), which increases cancer stem cell characteristics [18]. According to Zhu et al., CDC42 was enriched in colorectal cancer cells (CRC) by FOXD2-AS1 binding miR-185-5p, proposing that the FOXD2-AS1/miR-185-5p/CDC42 axis contributed to CRC progression [19]. likewise, Chen et al. identified that FOXD2-AS1 acted as a ceRNA for miR-363-5p to moderate transcription of S100A1, proposing that a FOXD2-AS1/miR-363-5p/S100A1 axis was involved in the progression of Nasopharyngeal carcinoma (NPC) [20]. Additionally, FOXD2-AS1 functioned as a molecular sponge of miR-485-5p to control KLK7 transcription, displaying that the FOXD2-AS1/miR-485-5p/KLK7 axis controlled the progression of Papillary thyroid cancer (PTC) [21]. In addition, FOXD2-AS1 may also activate Wnt/β-catenin signaling pathway؛ This pathway is active in many different types of tumors and plays an important role in controling the growth of cancer cells [22]. FOXD2-AS1 regulatory mechanisms are very complex and wide, including activation of LSD1 and EZH2, activating Wnt/β-catenin, TRIB3/Akt and NOTCH signaling pathways, inhibition of downstream gene transcription by competitively binding miRNAs and induction of the epithelial to mesenchymal transition (EMT) [16]. Taken together, upregulated FOXD2-AS1 normally acts as an unfavorable factor related to malignant clinicopathological features and shorter OS, suggesting that it could serve as a diagnostic and prognostic potential biomarker [16]. Moreover, transcription levels of CDK1 and CDC45 were elevated in primary AML non- M3 blood samples compared with G-CSF-mobilized healthy blood samples**.** CDK1 belongs to the serine/ threonine protein kinase family. Previous research has revealed that CDK1 is present in the cell cycle of numerous tumor-regulating cell adhesions [23] and may served as a potential biomarker for non-small-cell lung cancer(NSCLC) [24], CRC [25], breast cancer [26], and ovarian cancer [27]. In contrast, there is extremely little information about the function of CDK1 during tumorigenesis. CDK1 was identified to be overexpressed across numerous malignancies and is correlated with worse OS and RFS [28]. High expression levels are detected in some AML cases who did not respond to treatment, and increased nuclear CDK1 levels are associated with worse outcomes and lower complete remission (CR).Moreover, AML cases with relapse of disease have enhanced levels of CDK1 transcription [9]. In addition, upregulation levels of CDK1 were identified in leukemic cells with del(5q) [29]. Cytoplasmic loss of CDK1 was associated with therapy resistance in NSCLC cases [30]. Another research demonstrated that protein expression and enzymatic activity of CDK1, but not CDK2, significantly predicted distant metastasis in CRC [31]. Furthermore, targeting CDK1 increased FLT3-activated differentiation in AML cell lines because of C/EBPα which inhibits its differentiation-inducing function [32]. In line with our findings, we propose that CDK1 transcription and activity are related to several types of tumors. Regarding that CDK1 is can arrange with various cellular processes and regulate the cellular checkpoints, it is probable that any changes in the expression of CDK1 would affect the efficacy of therapeutic drugs.

CDC45 plays a key function in the initiation stage of DNA replication to ensure that chromosomal DNA is replicated only once per cell division [33]. CDC45 transcription is correlated with carcinogenesis and is valuable for tumor prognosis [34]. A previous study also showed that the level of CDC45 protein is steadily higher in several types of human cancer cells than in primary human cells and was detected as a proliferation-associated antigen [35]. According to some other studies, CDC45 is upregulated in some cancers, which led to the assumption that it is a leukemogenic gene [36, 37]. Moreover, CDC45 is responsible for DNA unwinding [37] and is recruited to the MCM2-7 complex to activate helicase [38]. Previous studies of solid tumors such as CRC, breast, and lung cancers have discovered increased levels of expression of CDC45, which stimulate the multiplication of cells [35, 39, 40]. Expression levels of CDC20 and CCNB1 at transcript were not statistically different between AML and healthy controls. Therefore, the previously reported deregulation of these genes could be due to regulatory mechanisms at the post-transcriptional level. Further research is needed to elucidate the mechanisms of such detected correlations. Based on our findings, these hub genes are crucial for AML progression and are associated with worse prognoses. In conclusion, the current study detected several key genes (Lnc-FOXD2AS1, CDK1 and CDC45), which could be served as novel and potential biomarkers of AML.

We emphasize the intricate and diverse roles of non-coding RNAs, particularly the dual functionality of antisense RNAs in cancer biology. Our study leverages a high-throughput data analysis methodology, including microarray data analysis. The GEO dataset supports community-derived reporting standards that specify provision of several critical study elements, including raw data, processed data, and descriptive metadata. The database not only provides access to data for tens of thousands of studies, but also offers various Web-based tools and strategies that enable users to locate the data. Besides, other methodologies such as RNAseq, and genomic analysis of cancer cells collectively contribute to our understanding of cancer, aiding in the diagnosis, treatment, and management of the disease. Moreover, the present AML dataset (GSE68172) acknowledges IL10RB-AS1 as a noteworthy candidate for further exploration, given its significant overexpression in AML according to our in-silico analysis. Nevertheless, this research is not without limitations. Some limitations in our research are listed as follows: We only used one dataset for the selection of deregulated genes. Although some datasets are similar, they fail to meet the selection criteria described above. To decrease bias in our work, we excluded these datasets. In conclusion, the differential expression of prognostic Lnc-FOXD2AS1 was validated by qRT-PCR, but more prospective investigations are needed to confirm its prognostic importance.

### Supplementary Information

**Additional file 1.**
