# Supplementary material for: Upregulation of lnc-FOXD2-AS1, CDC45, and CDK1 in patients with primary non-M3 AML is associated with a worse prognosis
Source: Blood Res. 2024 Feb 19;59(1):4. doi: 10.1007/s44313-024-00002-0 (PMC10903518; doi:10.1007/s44313-024-00002-0)
Supplement: Supplementary file 1 — Additional file 1. [file 44313_2024_2_MOESM1_ESM.docx]

**Supplementary Materials:**


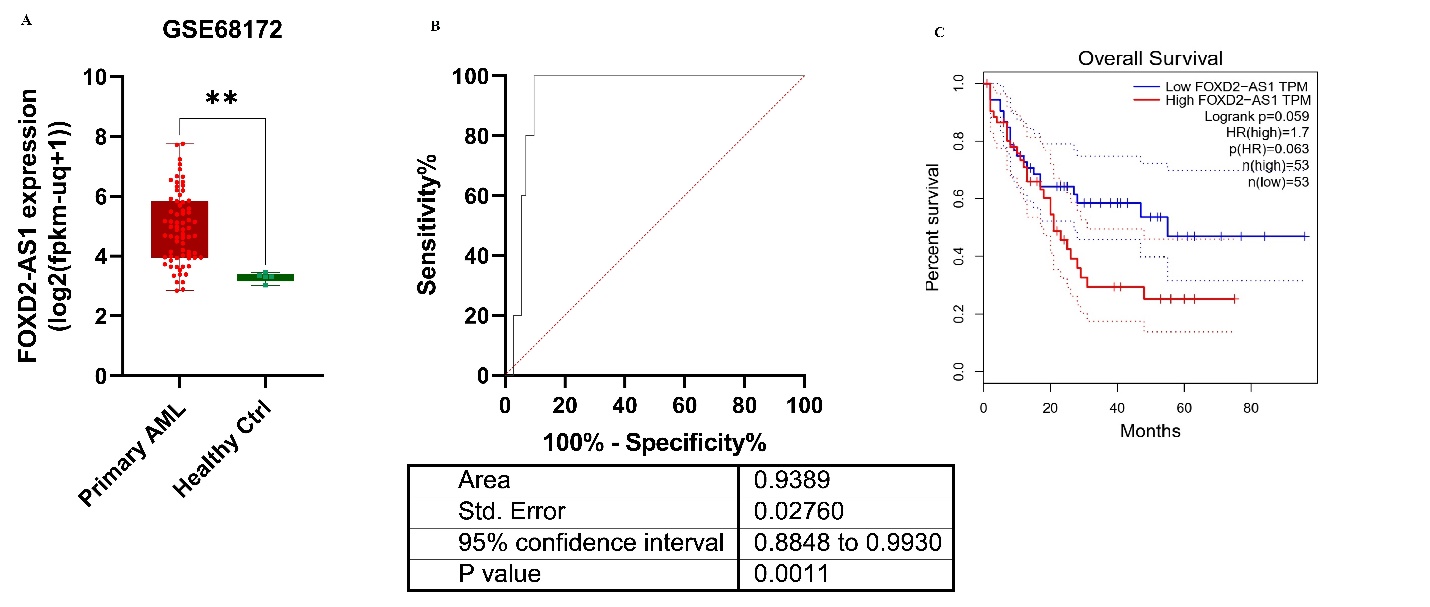


Figure **S1A**. Investigation of FOXD2-AS1 expression applying one GEO dataset (GSE68172) in primary AML and healthy controls. To measure statistical differences between categories, Wilcoxon matched-pairs signed-rank test, and Mann Whitney test were used.**S1B**. The application of FOXD2-AS1 overexpression has demonstrated its significance in differentiating primary AML cases from normal cases, as supported by the GSE68172 dataset.**S1C**. FOXD2-AS1 correlation with the overall survival of AML patients; TCGA-LAML cohort.

Table 1S**.** GO annotation, molecular function (a), cell component(b) and biological process(c)


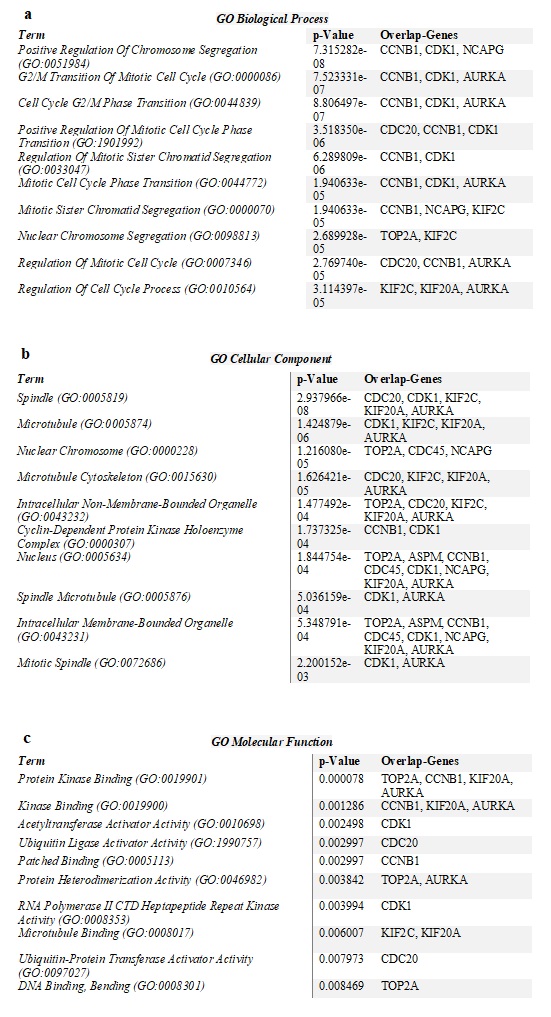


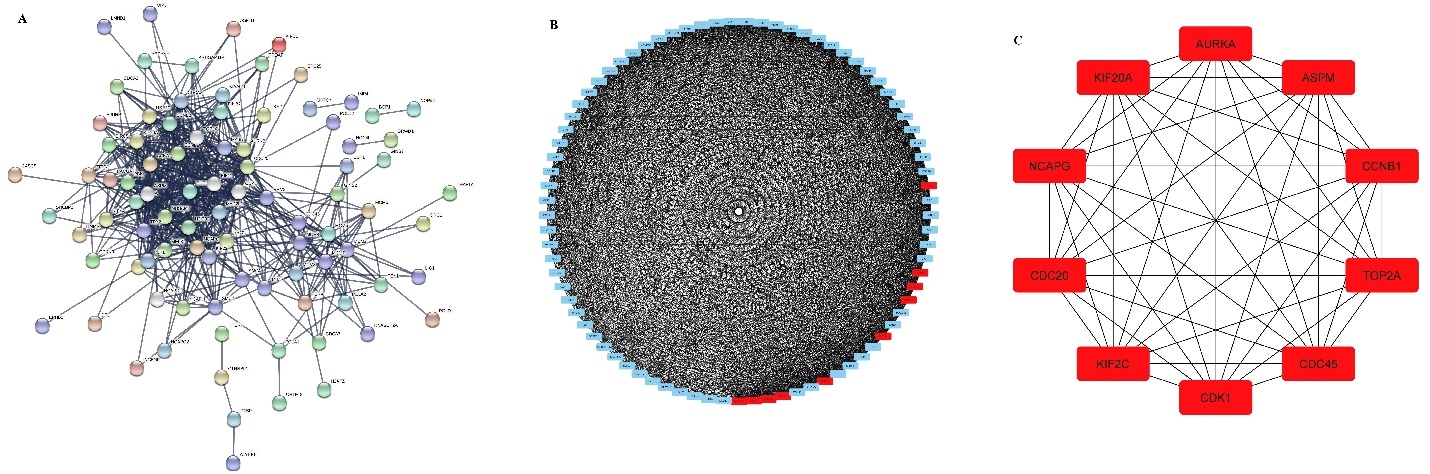


Figure 2S A-C. Construction of the PPI network pertaining to co-expressed genes associated with FOXD2-AS1. A. STRING B. Cytoscape. C. Identification of hub genes using the Cytohubba plugin in Cytoscape.


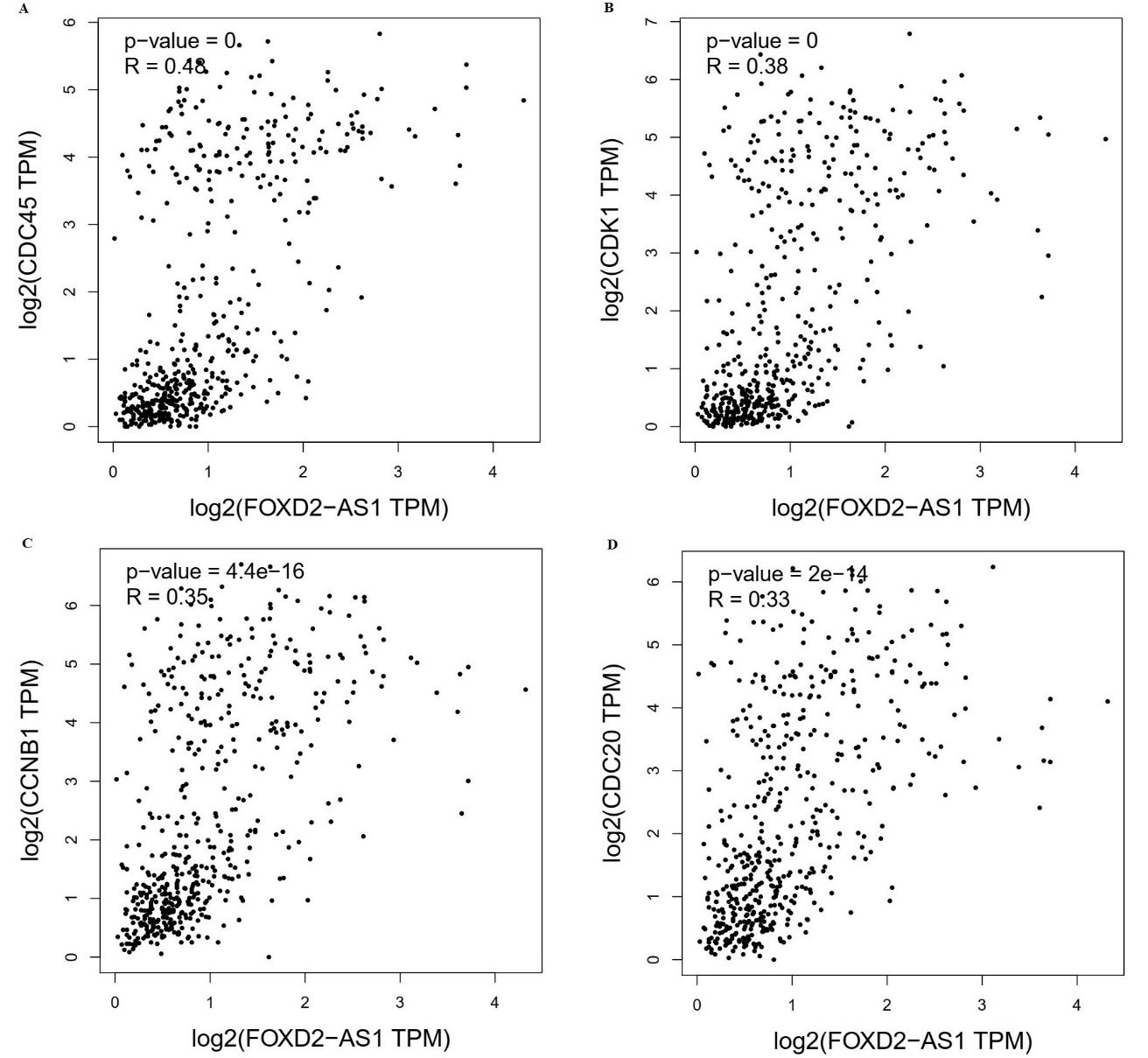


Figure 3S A-D. The association between FOXD2-AS1 and the transcription levels of CDC45(A), CDK1(B), CCNB1(C), and CDC20(D) was examined utilizing the TCGA-LAML patient cohort available in the GEPIA database.
